# Occupational Attributes and Occupational Gender Segregation in Sweden: Does It Change Over Time?

**DOI:** 10.3389/fpsyg.2020.00554

**Published:** 2020-03-26

**Authors:** Ingvill Bagøien Hustad, Johan Bandholtz, Agneta Herlitz, Serhiy Dekhtyar

**Affiliations:** ^1^Department of Clinical Neuroscience, Division of Psychology, Karolinska Institutet, Stockholm, Sweden; ^2^Department of Neurobiology, Care Sciences and Society, Aging Research Center, Karolinska Institutet, Stockholm, Sweden

**Keywords:** gender, segregation, longitudinal, occupational attributes, numerical, verbal, people, things

## Abstract

Sweden consistently ranks at the top of international assessments of gender equality, but paradoxically exhibits marked horizontal gender segregation in the labor market. By combining administrative and respondent-collected data, this study investigates whether occupational attributes are associated with sex distribution in Swedish occupations over a 10-year period between 2002 and 2011. Results show that the proportion of women was higher, on average, in occupations high in people orientation and verbal demands and lower in occupations high in things orientation and numerical demands. Mixed linear models showed a trend for desegregation during this period, as the proportion of women in people-oriented occupations has declined and a trend for an increase in the proportion of women in numerically demanding occupations was observed. Occupational attributes aid the understanding of gender segregation but patterns of segregation seem to change over time.

## Introduction

The segregation of men and women in Western labor markets is a topic discussed since the 1960s (Su et al., 2009). Ever since women started to enter the labor market in full force, it has become evident that the degree of female participation varies greatly between occupations, even within the same sectors (Ceci et al., 2009; Hegewisch et al., 2010; Ellingsæter, 2013). When discussing gender distribution in occupations, a distinction is typically made between two dimensions of segregation: vertical segregation, whereby men and women are clustered in different occupations in accordance with a hierarchical divide, and horizontal segregation, referring to a divide which occurs at the same level of occupational hierarchy and is instead based on the differences in occupational characteristics and attributes between the jobs occupied by men and women (Blackburn et al., 2001; Charles, 2003; Lippa et al., 2014).

The origins of horizontal sex segregation in occupations have been extensively debated and different perspectives have been voiced in both sociology and psychology. For example, gender stereotypes and their implications for educational and career choices have been debated (Correll, 2001; Charles and Bradley, 2009). Some research has shown that boys tend to have a higher self-assessment and sense of self-efficacy in mathematics than girls – also when matched on performance (Correll, 2001; Else-Quest et al., 2010). Cross-national studies have suggested that people implicitly associate men, rather than women, with scientific occupations, but that the strength of these associations is weaker in countries where the representation of women in scientific fields is higher (Miller et al., 2015). Stereotypes have also been proposed to interact with new norms of self-expression and employment structures in post-industrialized contexts and might thus help explain why horizontal sex segregation is more substantial in countries high in economic prosperity and material safety (Charles and Bradley, 2009; Barone, 2011).

From a psychological perspective, one’s cognitive abilities, sense of self-efficacy and interests may guide one’s occupational choice, as suggested in social cognitive career theory (Lent et al., 1994). Previous studies have found gender differences in cognitive abilities where men have a slight advantage in complex mathematics and visuospatial tasks (Guiso et al., 2008; Else-Quest et al., 2010; Reilly, 2012; Hyde, 2014; Weber et al., 2014), while girls outperform boys on verbal measures and episodic memory (Reilly, 2012; Weber et al., 2014). Some researchers have questioned the magnitude and implications of such cognitive differences (Ceci et al., 2014; Hyde, 2014). A recent study has shown that gender differences in academic performance, which largely mirror gender differences in cognitive abilities, appear to have a long-lasting impact on individual life outcomes by affecting women and men’s career choices (Dekhtyar et al., 2018).

While occupational gender segregation arises due to an interplay between multiple individual and environmental factors, it does not remain constant, but instead evolves over time. Thus, there has been a trend for gender desegregation in most industrialized labor markets since the 1960s (England and Li, 2006; Barone, 2011). However, the rate of desegregation has been unevenly distributed between occupations, exhibiting a trend toward stagnation in recent years (Tomaskovic-Devey et al., 2006), and mirroring similar developments as in gender segregation in education (England and Li, 2006; Ceci et al., 2009; Charles and Bradley, 2009; Barone, 2011). Sweden might be especially well-suited for investigating the evolution of occupational gender segregation, as it has been widely noted for being at the forefront of gender egalitarianism according to international assessments (World Economic Forum, 2013, 2014, 2015, 2016). Paradoxically, however, the Swedish labor market remains one of Europe’s most horizontally segregated, a development largely attributed to a disproportionate share of Swedish women joining already predominantly female-dominated occupations during a rapid increase in female labor force participation between 1970 and 1990’s (Melkas and Anker, 1997). It is therefore of much interest to investigate the recent changes in occupational gender segregation in a Swedish environment where measures to achieve gender equality have been predominantly targeted vertical, but not horizontal segregation.

One way of approaching the study of trends in occupational gender segregation is to identify salient attributes that characterize different occupations and then examine if sex distribution across the occupational landscape varies in accordance with these occupational prerequisites. Earlier research has shown that a dimension of people-things orientation, an occupational orientation toward either people or mechanical objects and systems, is strongly associated with occupational sex distribution: women more often work in people-oriented occupations, and men in things-oriented ones (Ceci et al., 2009; Su et al., 2009; Barone, 2011; Lippa et al., 2014). Further, based on the previously mentioned research on cognitive gender differences (Guiso et al., 2008; Reilly, 2012; Weber et al., 2014; Dekhtyar et al., 2018), it may be interesting to investigate whether an occupation’s perceived degree of verbal vs. numerical cognitive demands is associated with its sex distribution. Finally, because of changes in the patterns of occupational gender segregation, the emergence of new labor markets, and evolving employment structures, it is of further interest to consider whether the association between occupational attributes and the sex distribution has changed over time.

The purpose of the current study is to investigate the patterns of occupational gender segregation in Sweden from the perspective of occupational prerequisites. Specifically, we examine if an occupation’s level of things and/or people orientation is associated with the proportion of women working in that occupation. We also examine the association between numerical and/or verbal demands in occupations and the proportion of women. Furthermore, we investigate whether the association between occupational attributes and female share in occupations has evolved differentially over time. For instance, has the share of women been in numerically demanding occupations changed faster than in occupations characterized by other types of attributes? This study contributes to the previous literature on horizontal gender segregation and its temporal evolution (see for instance Nermo, 2000) in several important ways. First, we characterize occupations according to attributes, which has the advantage of going beyond the classifications of fields of study usually deployed in research on gender segregation in education (eg. STEM or humanities etc.). This way, we offer another perspective into factors contributing to gender segregation, while, importantly, also tracking its development over time in Sweden, a setting paradoxically defined by low vertical and high horizontal segregation. Finally, we also have the benefits of full-population data to compute occupational sex ratios in a wide spectrum of occupations (and not just in broad industry sectors).

Based on the literature, four hypotheses have been formulated: (1) there will be a positive association between the proportion of women and occupations’ degrees of people orientation and verbal demands, (2) there will be a negative association between the proportion of women and occupations’ degrees of things orientation and numerical demands, (3) over time, the proportions of women will have increased on average in all occupations, and (4) the rate of change over time will have differed between occupations characterized by the four different attributes (people orientation, things orientation, verbal demands and numerical demands).

## Materials and Methods

### Study Population

The material used in the current study is based on both already existing administrative data as well as collected data from respondents. Administrative data were extracted from the Swedish bureau of statistics, Statistics Sweden (SCB). The unit of analysis in the study is all 355 occupational titles in Sweden. The titles are derived from a categorical classification of professions (SSYK96), which identifies separate occupational titles primarily in accordance with the field of study required to enter a given position (see Supplementary Table S1 for a list of all occupational titles as well as all data used in the study).

### Dependent Variable

The proportion of women in each of the 355 occupations between 2002 and 2011 serves as the dependent variable. It was computed by Statistics Sweden using the entire working population of Swedish residents aged 18–64 throughout the time period. Over the examined 10-year period, the average population was *N* = 4,460,360 with a span of 4,361,500–4,625,900. The dependent variable reflects the gender distribution in each occupation, but not the actual number of individuals working in it. In other words, one occupation might show a large degree of segregation, but the underrepresented gender can reflect either a negligible or substantial absolute number of workers employed in that profession.

### Independent Variables

Data collected from respondents was used to generate independent variables (see procedure below). For the independent variables, four occupational attributes were included: numerical demands, verbal demands, things orientation and people orientation. The four occupational attributes constitute two categories. One describes occupational demands, that is, the nature of the cognitive skills necessary to perform the most dominant task in a given occupation. The other category describes occupational orientation, that is, what one works with or manipulates in a given occupation.

People orientation describes to what extent the main task in a given occupation involves working with people and interpersonal relations in various situations. Examples of highly people-oriented occupations may be nurses, psychologists and teachers. Things orientation describes to what extent the main task in a given occupation requires working with and/or manipulating things, meaning materials, gadgets and objects. Examples of highly things-oriented occupations may be mechanics, carpenters and tailors. Verbal demands describes the degree of verbal tasks and assignments performed in a given occupation. This pinpoints central cognitive skills relating to communication skills and language proficiency. Examples of occupations high in verbal demands may be linguists, lawyers and TV- and radio presenters. Numerical demands describes the degree of numerical tasks and operations performed, thus pinpointing central cognitive skills required to work in this occupation. Examples of occupations high in numerical demands may be statisticians, engineers and computer programmers.

### Procedure

To obtain four measures of occupational attributes described above, student respondents rated 355 occupational titles on each attribute. Data from 15 respondents was collected, where each of the 355 occupations were rated on a 7-point Likert scale (1 = lowest value, 7 = highest value) for each of the four occupational attributes. Ratings were collected during individual sessions lasting approximately 1.5–2.5 h, including short breaks. The respondents were selected to represent the knowledge and understanding of the group of individuals who are to begin their occupational career. In order to mimic the initial screening of the occupational landscape, the respondents were instructed to rate the occupational titles according to their own spontaneous impression of the main task performed in the given occupation. Respondents constituted a convenience sample recruited through advertisement via social media channels and word of mouth. All respondents, 8 women and 7 men aged 22–29 years (*M* = 25.86, SD = 2.26 years), were Swedish citizens with Swedish as their mother tongue. Ten were active students at different universities and other institutions for higher education in the Stockholm area. Further, four were either in between studies or recently graduated, and one respondent had no higher education. The fields of study varied between the respondents, ranging from humanities to science. Written informed consent was obtained from all respondents who were blind to the aim of the study and were informed that they could discontinue the survey at any time and still receive the compensation of two cinema tickets.

### Statistical Analyses

The first part of the empirical strategy involved preparation of evaluator-based ratings for further analyses. Data from all respondents were used. Raw scores were standardized for each participant and used to calculate mean Z-scores for each occupation and occupational attribute. Using the STATA plug-in IRA, several measures of inter-rater-agreement (rwg; awg) were calculated to assess the consistency of respondent ratings.

### Descriptive Analyses

All descriptive analyses omit the time perspective and instead explore the average proportion of women in each occupation over the period 2002–2011. Correlations were calculated using Pearson’s *r*, assessing the associations between the four attributes and the average proportion of women working in the corresponding occupations in 2002–2011. A correlation matrix between the four attributes was also calculated to investigate the potential multicollinearity (Table 1) between the independent variables. For regression analyses, four models were fitted, one for each occupational characteristic’s association with the average proportion of women working in that occupation. Because of apparent multicollinearity, no multiple linear regression analyses were utilized, and only simple linear regression analyses were performed.

**TABLE 1:** Correlation matrix.

Occupational attribute	People orientation	Things orientation	Verbal demands	Numerical demands	Proportion of women
People orientation	1				0.625**
Things orientation	−0.740**	1			−0.502**
Verbal demands	0.859**	−0.824**	1		0.518**
Numerical demands	−0.158**	−0.053	0.003	1	−0.136*

*p < 0.05, **p < 0.001. The coefficients reflect the correlation between occupational attributes and the average proportion of women in all occupations between 2002 and 2011, as well as correlations between ratings of occupational attributes.

### Multilevel Modeling

Mixed linear models (MLM) (Duncan et al., 2013) were used to explore the following questions: (1) how has the average proportion of women on the Swedish labor market changed between 2002 and 2011? and (2) has this change over time been uneven depending on occupations’ occupational attributes? These questions correspond to the overall study hypotheses 3 and 4 (for details, see section “Introduction”). For the multilevel modeling analyses, the proportion of women in each occupation and year is the dependent variable. Independent variables are occupations’ rated levels of numerical demands, verbal demands, things orientation and people orientation, as well as time (years between 2002 and 2011). For our multilevel models, occupations (unit of analysis in the study) were nested in time. We assumed that the change in female proportion will follow a linear trend over time, although the exact intercepts and slopes were allowed to vary across the 355 occupational titles (level one) and 10 years (level two). This was enabled by defining random effects for individual occupation and follow-up time. Unstructured covariance matrix was assumed in the analysis. The parameters of interest from the MLM models are the fixed effects for time (designating average change in the proportion of women in all Swedish occupations between 2002 and 2011), the fixed effect for occupational attribute (designating the average proportion of women in occupations with a given attribute at the beginning of the study period), and the interaction term between occupational attribute and time (designating the mean rate of change in the proportion of women between 2002 and 2011 in occupations defined by a given attribute).

## Results

The 355 occupations were rated by the 15 respondents on each of the four attributes: numerical demands, verbal demands, things orientation, and people orientation. To estimate the internal consistency of the responses, several inter-rater-agreement indexes were computed. Conventional rwg estimates ranged between 0.95 and 0.99, which is expected in this relatively large sample. Following Brown and Hauenstein (2005), we additionally computed awg(j) index which is less susceptible to sample size and scale used. Resulting indices ranged between 0.61 and 0.65 depending on assessed occupational attribute, which is comparable to conventionally adopted threshold of 0.70 used to indicate acceptable inter-rater agreement.

The correlations between the components of occupational attributes are presented in Table 1. Given the sizeable inter-correlations obtained, our empirical strategy was adjusted to only examine bivariate associations between occupational attributes and the outcome in linear regression analysis, whereas for the MLM, we considered the effects separately for the two dimensions (orientation vs. demands). As such, the four attributes were never entered in the same model to avoid multicollinearity.

Univariable linear regressions were calculated to investigate if the average proportion of women in Swedish occupations between 2002 and 2011 varied in accordance with occupational attributes (see Table 2 for regression results). All estimates were statistically significant. With an increase of one standard deviation in people orientation, the unstandardized beta-coefficient suggests that the average proportion of women increased by 20.8%. An increase of one standard deviation in things orientation yielded a decrease of 18.8% in the proportion of women. For verbal demands, the proportion of women increased by 17.9% for each increase in standard deviation. Lastly, an increase of one standard deviation in numerical demands showed a 5.1% decrease in the proportion of women.

**TABLE 2:** Univariable linear regressions.

Occupational attribute	β	*R*^2^	*p*
People orientation	0.208	0.390	<0.001
Things orientation	−0.188	0.252	<0.001
Verbal demands	0.179	0.268	<0.001
Numerical demands	−0.051	0.019	0.010

*Results from univariable linear regression analyses for each of the four occupational attributes. Dependent variable: average proportion of women in 2002–2011. Based on four separate linear models. Abbreviations: β, unstandardized beta coefficient, R^2^, coefficient of determination; p, p-value.*

**FIGURE 1:**
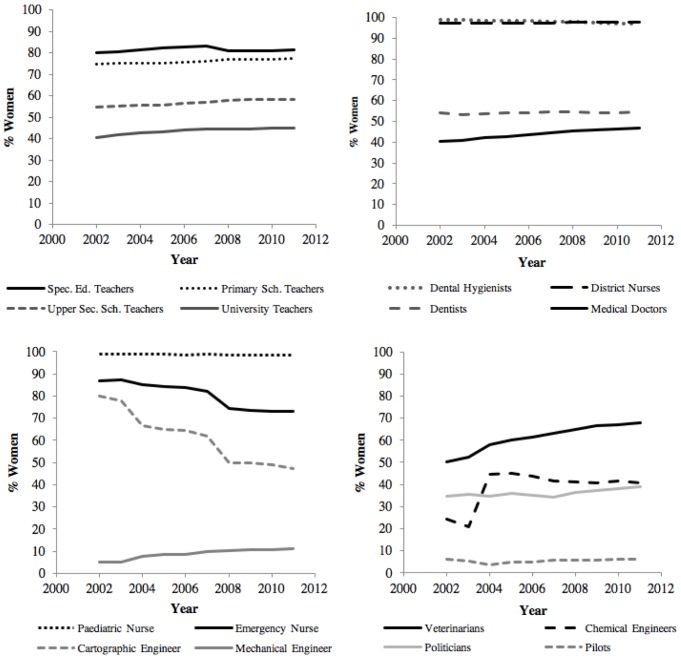
Trajectories of change in the proportion of women over time. Selected occupations during years 2002–2011. These occupations serve as examples of the considerable heterogeneity in both initial proportions of women in 2002, as well as the subsequent developmental trajectories.

Between 2002 and 2011, some occupations showed an increase in the proportion of women, some had an unchanged proportion and still others showed a decline (see Figure 1 for a graphical depiction of selected trajectories over time). Even when examining similar occupations, at times large differences in both gender distributions and trajectories emerge. Given these examples, we next examined the interactions between occupational attributes and time to establish whether the proportion of women changed at a significantly different pace over time depending on occupational attributes.

The estimates for fixed effects (see Table 3) showed a significant effect of time, where the overall proportion of women in the labor market increased by 3.1% over 10 years. There was a significant main effect for people orientation, with higher proportions of women working in people-oriented occupations – a finding mirrored in univariable linear models above. However, there was no significant main effect for things orientation on the proportion of women – potentially an artifact of collinearity between people and things orientation (*r* = −0.740). A significant negative interaction between people orientation and time was found. This indicates that women’s relative participation in occupations with high scores on people-orientation was significantly reduced relative to the average rate of change in the proportion of women over the time period. There was no significant interaction between things orientation and time. Random effects of slope, intercept and residual were all significant at the 95% level, suggesting some heterogeneity around the mean estimates reported above.

**TABLE 3:** Mixed linear model: people and things orientation.

Fixed parameters	β	*p*	CI (95%)	
			**Lower**	**Upper**
Time	0.031	< 0.001	0.022	0.040
Intercept	0.374	< 0.001	0.349	0.398
People	0.196	< 0.001	0.154	0.238
People × time	–0.018	0.024	–0.034	–0.002
Things	–0.025	0.307	–0.073	0.023
Things × time	–0.015	0.094	–0.033	0.003
**Random parameter (Variance)**				
Slope (time)	0.007		0.006	0.008
Intercept	0.055		0.047	0.063
Residual	0.001		0.001	0.001

*Dependent variable: proportion of women in each occupation and every year between 2002 and 2011. Time was scaled 0–1 with equal spacing for each year, allowing for coefficient to be interpreted as change over 10 years. Abbreviations: β, unstandardized beta coefficient, p, p-value. CI, confidence interval.*

Detailed results for the association between verbal-numerical demands and occupational gender occupation over time are in Table 4. The fixed effects for the intercept and the main effect of time are the same as reported in MLM Model 1. There was a statistically significant positive main effect of verbal demands on the proportion of women, implying higher proportions of women, on average, in verbally demanding occupations. There was also a significant negative main effect for numerical demands, implying lower proportions of women in occupations high in these demands. Both main effects are consistent with earlier-estimated bivariate linear associations (Table 2). No significant interaction effect was found between verbal demands and time. This indicates that women’s participation in occupations rated as verbally demanding did not vary relative to the average pace of change during this period. A positive interaction effect between numerical demands and time approached statistical significance (*p* = 0.058). This indicates that women’s relative participation in numerically demanding occupations may have increased at a faster pace relative to the average rate of change over this 10-year period. Examining random effects indicated that the linear growth rates of women’s participation in occupations between 2002 and 2011 varied significantly between occupations.

**TABLE 4:** Mixed linear models: verbal and numerical demands.

Fixed parameters	β	*p*	CI (95%)	
			Lower	Upper
Time	0.031	< 0.001	0.022	0.040
Intercept	0.373	< 0.001	0.347	0.399
Verbal	0.179	< 0.001	0.148	0.212
Verbal × time	–0.002	0.789	–0.012	0.010
Numerical	–0.057	0.001	–0.092	–0.023
Numerical × time	0.011	0.058	0.000	0.024
**Random parameter (Variance)**				
Slope [time]	0.007		0.006	0.008
Intercept	0.063		0.055	0.074
Residual	0.001		0.001	0.001

*Dependent variable: proportion of women in each occupation and every year between 2002 and 2011. Time was scaled 0–1 with equal spacing for each year. Abbreviations: β, unstandardized beta coefficient, p, p-value. CI, confidence interval.*

## Discussion

This study found that the Swedish labor market exhibits marked horizontal gender segregation but also that the patterns of said segregation varied over a 10-year period between 2002 and 2011. Certain occupational attributes were associated with occupational gender distribution in Sweden. There was a positive relationship between female representation and the degree of people orientation and verbal demands, with a larger proportion of women working in occupations ranked high on these attributes. Fewer women, relatively speaking, were represented in occupations high in things orientation and numerical demands, although the association for the latter was weaker. Between 2002 and 2011, the average proportion of women in the Swedish workforce increased by 3.1%. The rate of increase was reduced in highly people-oriented occupations. For numerically demanding occupations, there was a trend toward a larger increase in the proportion of women than the average gain over the time period, although this result only approached statistical significance.

Our findings indicate that perceptions of occupational prerequisites are an important aspect in order to describe patterns of occupational gender segregation. Regarding the verbal-numerical dimension, previous research on cognitive gender differences has shown a slight male advantage in complex mathematics and visuospatial tasks and a female advantage in verbal skills (Reilly, 2012; Weber et al., 2014). Recent research suggests that such differences can affect later gender segregation in educational and occupational choices (Dekhtyar et al., 2018). Our findings are consistent with these patterns in the sense that women were overrepresented in verbally demanding occupations and somewhat underrepresented in numerically demanding ones. However, the current results also show that this occupational pattern may be changing over time in Sweden, as a trend for a slight increase in the proportion of women in numerically demanding occupations was noted. When considering cognitive differences, this is also in line with research showing that women’s mathematical performance increases more than men’s in economically prosperous and gender equal countries (Guiso et al., 2008; Weber et al., 2014).

The current study is based on the time period 2002–2011 and, when considering change over time, we did not find a similar trend for a stagnation of desegregation that earlier research has showed for preceding decades (England and Li, 2006; Tomaskovic-Devey et al., 2006; Barone, 2011). Using data from a 10-year period made it possible to track both the average change over time, but also to identify heterogeneity in the trajectories and interactions with occupational attributes. Despite an occupation’s people orientation being strongly correlated with the proportion of women, the current study shows that the proportion of women in people-oriented occupations showed a reduced pace of change. Considering a trend for a faster growth of female representation in numerically demanding occupations, this could mean that despite the vast gender segregation seen today, Sweden might slowly be heading toward some occupational desegregation. The current results show that patterns of segregation can change over time, which alters the predictive value of specific variables. In other words, despite people orientation being strongly associated with the proportion of women today, our findings suggest that this relationship might weaken with time as women leave and/or men enter these occupations to varying extent.

Occupational gender segregation is influenced by many factors. For instance, cross-national studies have shown that countries that are ranked lower on international measures of gender equality show a higher representation of women in STEM-fields (Charles and Bradley, 2009). The suggested explanation for this is that women have more to gain from entering these occupations in terms of status and economic resources (Charles and Bradley, 2009). Finland is one of the few countries where girls have reached parity with boys on measures of mathematical abilities (Reilly, 2012), although the country’s gender gap in math affinity is one of the largest in international comparison (Charles and Bradley, 2009). It might be that, because of norms of self-expression in post-industrialized countries, girls are less likely to express affinity for math because it is still not in line with current gender schemas (Charles and Bradley, 2009). Another aspect worth noting is gender representation. The representation of women in science fields leads to weaker implicit and explicit gender stereotypes about scientists, in turn leading to even more women entering these fields in the future (Miller et al., 2015).

The current study has the advantage of including all Swedish occupations between 2002 and 2011, as classified by the Swedish Statistics Bureau, where the entire working population between 18–64 years of age is included. However, there are also some limitations. First, the limited number of respondents for the estimation of the occupational attributes, and the fact that these respondents were university students, and not work experts, could be a source of limitation. The reason for selecting students was to mimic the knowledge and the understanding of individuals who are about to embark on an occupational career. Our intention was to replicate the initial screening of the occupational landscape by the future workers who are supposedly looking to match their own skills with the perceived attributes of the careers in question (hence, our request that the students use their initial, spontaneous judgment). Importantly, the estimation of verbal and numerical demands of occupations used here, has previously (Dekhtyar et al., 2018) been found to be related to an existing classification (O^∗^NET) of United States occupational titles (National Center for O^∗^Net Development, 2016). We decided to use our measure of numerical and verbal cognitive demands as not all Swedish occupations could be matched to a corresponding United States equivalent. The internal consistency was high, indicating high rating agreement among our respondents. It is not clear whether a larger number of respondents would have resulted in a more valid or reliable measure, but if it did, the present results would be an underestimation of the true associations between the independent and dependent variables. Taken together, we believe that the ratings of occupational demands (verbal, numerical) and orientation (people, things) are reliable and valid, and correspond well to the perception of young individuals’ beliefs about the occupations they are about to select. Secondly, this study uses occupational titles as the unit of analysis, and as such, individual workers’ abilities or interests are unobserved here. We observed considerable collinearity between people orientation and verbal demands, which is expected, as working with people is almost synonymous with using one’s verbal abilities. Nevertheless, it was our explicit aim to collect measures of both occupational demands and orientation, as they likely capture different facets of occupational attributes. A time perspective longer than the 10 years investigated here could help better delineate the temporal effects, particularly with respect to structural shocks, policy changes, crises, or other period effects. Finally, the aim of this study was descriptive and was meant to establish association, rather than causation between occupational attributes and changes in horizontal gender segregation. Future studies that attempt to get closer to the causal effect of occupational attributes as well as individual abilities and interests, on career choices, are needed. While experimental approaches may be limited for these questions, techniques using propensity score matching, inverse-probability weighting, or Mendelian randomization approaches could prove especially useful in the future.

In sum, this study shows that occupational attributes aid the understanding of occupational gender segregation. The patterns of segregation change over time, and a growth curve analysis shows a tentative trend toward some desegregation in Sweden.

## Data Availability Statement

All datasets generated for this study are included in the article/Supplementary Material.

## Author Contributions

Substantial contribution to the conception of design of the work: IH, JB, and SD. Acquisition of data: SD, IH, JB, and AH. Analysis: IH, JB, and SD, interpretation of data; IH, JB, AH, and SD. Drafting of the manuscript or revising it critically for important intellectual content: JB, IH, AH, and SD. Final approval of the version to be published: IH, JB, AH, and SD. Agreement to be accountable for all aspects of the work: IH, JB, AH, and SD.

## Conflict of Interest

The authors declare that the research was conducted in the absence of any commercial or financial relationships that could be construed as a potential conflict of interest.

## Supplementary Material

TABLE S1 Data used in longitudinal analysis. SSYK: occupational title code; women: proportion of women in a given occupation/year; numerical: ranking of a given occupation in terms of numerical demands; verbal: ranking of occupations in terms of verbal demands.
